# Tocotrienols-induced inhibition of platelet thrombus formation and platelet aggregation in stenosed canine coronary arteries

**DOI:** 10.1186/1476-511X-10-58

**Published:** 2011-04-14

**Authors:** Asaf A Qureshi, Charles W Karpen, Nilofer Qureshi, Christopher J Papasian, David C Morrison, John D Folts

**Affiliations:** 1 Department of Basic Medical Science, School of Medicine, 2411 Holmes Street, University of Missouri, Kansas City. MO 64108, USA; 2 Advanced Medical Research, 8251 Raymond Road, Madison, Wisconsin, 53719, USA; 3 Prairie Cardiovascular Consultants, 619 East Mason Street, Suite 4P57, Springfield, IL 62701, USA; 4 Department of Pharmacology/Toxicology, 2464 Charlotte Street, University of Missouri, Kansas City, MO 64108, USA; 5 Department of Medicine, Cardiovascular Medicine Section, 2537 Chamberlain Av. Room A, Madison Wisconsin, 53705, USA

## Abstract

**Background:**

Dietary supplementation with tocotrienols has been shown to decrease the risk of coronary artery disease. Tocotrienols are plant-derived forms of vitamin E, which have potent anti-inflammatory, antioxidant, anticancer, hypocholesterolemic, and neuroprotective properties. Our objective in this study was to determine the extent to which tocotrienols inhibit platelet aggregation and reduce coronary thrombosis, a major risk factor for stroke in humans. The present study was carried out to determine the comparative effects of α-tocopherol, α-tocotrienol, or tocotrienol rich fraction (TRF; a mixture of α- + γ- + δ-tocotrienols) on *in vivo platelet thrombosis *and *ex vivo *platelet aggregation (PA) after intravenous injection in anesthetized dogs, by using a mechanically stenosed circumflex coronary artery model (Folts' cyclic flow model).

**Results:**

Collagen-induced platelet aggregation (PA) in platelet rich plasma (PRP) was decreased markedly after treatment with α-tocotrienol (59%; ***P ***< 0.001) and TRF (92%; ***P ***< 0.001). α-Tocopherol treatment was less effective, producing only a 22% (***P ***< 0.05) decrease in PA. Adenosine diphosphate-induced (ADP) PA was also decreased after treatment with α-tocotrienol (34%; ***P ***< 0.05) and TRF (42%; ***P ***< 0.025). These results also indicate that intravenously administered tocotrienols were significantly better than tocopherols in inhibiting cyclic flow reductions (CFRs), a measure of the acute platelet-mediated thrombus formation. Tocotrienols (TRF) given intravenously (10 mg/kg), abolished CFRs after a mean of 68 min (range 22 -130 min), and this abolition of CFRs was sustained throughout the monitoring period (50 - 160 min).

Next, pharmacokinetic studies were carried out and tocol levels in canine plasma and platelets were measured. As expected, α-Tocopherol treatment increased levels of total tocopherols in post- vs pre-treatment specimens (57 vs 18 μg/mL in plasma, and 42 vs 10 μg/mL in platelets). However, treatment with α-tocopherol resulted in slightly decreased levels of tocotrienols in post- vs pre-treatment samples (1.4 vs 2.9 μg/mL in plasma and 2.3 vs 2.8 μg/mL in platelets). α-Tocotrienol treatment increased levels of both tocopherols and tocotrienols in post- vs pre-treatment samples (tocopherols, 45 vs 10 μg/mL in plasma and 28 vs 5 μg/mL in platelets; tocotrienols, 2.8 vs 0.9 μg/mL in plasma and 1.28 vs 1.02 μg/mL in platelets). Treatment with tocotrienols (TRF) also increased levels of tocopherols and tocotrienols in post- vs pre-treatment samples (tocopherols, 68 vs 20 μg/mL in plasma and 31.4 vs 7.9 μg/mL in platelets; tocotrienols, 8.6 vs 1.7 μg/mL in plasma and 3.8 vs 3.9 μg/mL in platelets).

**Conclusions:**

The present results indicate that intravenously administered tocotrienols inhibited acute platelet-mediated thrombus formation, and collagen and ADP-induced platelet aggregation. α-Tocotrienols treatment induced increases in α-tocopherol levels of 4-fold and 6-fold in plasma and platelets, respectively. Interestingly, tocotrienols (TRF) treatment induced a less pronounced increase in the levels of tocotrienols in plasma and platelets, suggesting that intravenously administered tocotrienols may be converted to tocopherols. Tocotrienols, given intravenously, could potentially prevent pathological platelet thrombus formation and thus provide a therapeutic benefit in conditions such as stroke and myocardial infarction.

## Background

Americans are often encouraged to consume more fruits and vegetables, and eat less red meat and dairy products, in order to lower total and LDL-cholesterol levels and reduce the risk of developing atherosclerosis, platelet mediated coronary thrombosis, myocardial infarctions, and strokes [1,2]. It is now known that oxidized LDL-cholesterol is a much greater risk factor for the development of atherosclerosis than its non-oxidized precursor [3-7]. Dietary antioxidants, such as vitamin E (α-tocopherol), have been shown to prevent LDL oxidation and limit hypercholesterolemia in humans [8,9], and it has been proposed that Vitamin E can prevent vascular thrombosis and atherosclerosis [8-11]. A number of studies have suggested that dietary intake of small doses of α-tocopherol reduces the risk of developing coronary atherosclerosis [9,10], although this has not been a universal finding [12,13].

The inhibitory effects of α-tocopherol on platelet function *in vivo *and *ex vivo *have been well established [14-18], but inhibition of platelet aggregation by α-tocopherol *in vitro *requires non-physiologically high levels of α-tocopherol [14]. It is not surprising, therefore, that the results of *in vivo *and *in vitro *studies are often in conflict when studying the effects of α-tocopherol on platelet aggregation [14-18]. The mechanism by which α-tocopherol reduces thrombosis and atherosclerosis is not well defined [7,8], though a number of possible mechanisms have been proposed; the antioxidant properties of α-tocopherol are often implicated [8]. Recently, several investigators have reported that tocotrienols (unsaturated vitamin E) have 10- to 20-fold greater antioxidant activity than α-tocopherol (vitamin E), and provide more efficient protection against certain free radical-related diseases than α-tocopherol [19-28].

**Figure 1:**
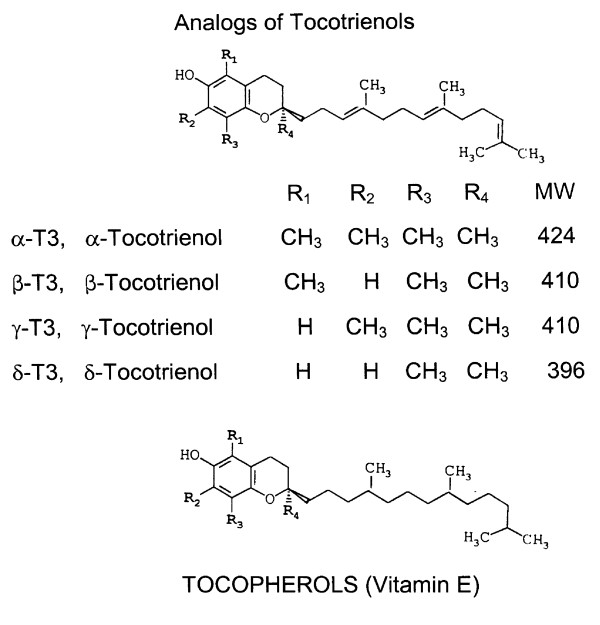
**Chemical structures of isomers of tocopherols and tocotrienols used in this study**.

The positive effects of tocotrienols (unsaturated vitamin E; Figure 1) as anti-inflammatory, antioxidant, anticancer (anti-proliferative), hypocholesterolemic, and neuroprotective agents in animal and humans have been confirmed by several investigators [23,29-39]. Tocotrienols are naturally-occurring farnesylated unsaturated analogs of α-, β-, γ- and δ-tocopherols (Figure 1) isolated from barley, rice bran, and annatto seeds [21]. The Folts' arterial injury model of stenosis and thrombosis provides a system for evaluating the effects of various natural products *in vivo *and *ex vivo *on platelet aggregation and thrombus formation [40]. In the experiments described in this manuscript, we determined the effects of α-tocopherol, α-tocotrienol, and a purified tocotrienol rich fraction from palm oil without α-tocopherol (TRF) on both *in vivo *platelet thrombus formation and *ex vivo *platelet aggregation in the Folts' model using anesthetized dogs with mechanically stenosed circumflex coronary arteries (MSCCA). The concentrations of tocopherols and tocotrienols were also estimated in pre-dose and post-dose samples of plasma and platelets.

## Materials and methods

### Materials

Soluble equine tendon type I collagen and adenosine diphosphate (ADP) were purchased from Chrono-Log Corp (Havertown, PA, USA). Manual platelet counting was performed using the Unipette System. Dogs were obtained from the Medical School Facility of the University of Wisconsin, Madison, 53706, USA.

The investigation conforms with the guidelines for the care and use of laboratory animals by the US National Institute of Health (NIH publication 85-23, revised 1996), and the University of Wisconsin Research Animal Resource Center. The protocol was reviewed and approved by the University of Wisconsin-Madison College of Agricultural and Life Sciences Animal Care and Use Committee.

#### Canine model and surgical preparation

Ten dogs were anesthetized, the chest opened and the heart exposed. The left circumflex coronary artery was dissected out and an electromagnetic flow probe placed circumferentially as previously described [40]. Distal to the flow probe, the artery was clamped three times with a special surgical clamp to produce intimal and medial damage, and a plastic cylinder of appropriate diameter was then placed circumferentially to produce a 70% reduction in internal arterial diameter [40]. Platelet-mediated thrombi periodically form at the site of stenosis, and embolize distally. These thrombi were monitored by the flow probe as cyclic flow reductions (CFRs) in coronary blood flow (40). The size and frequency of these CFRs is directly related to the level of *in vivo *platelet activity [40]. The average body weight of the dogs was 25 ± 0.6 kg for all groups.

### Preparation of TRF (mixture of α- + γ- + δ-tocotrienols) free of α-tocopherol from palm oil by flash chromatography

Silica gel (Merck, 230-400 mesh, 60 Å, 500 g) suspended in 1000 mL of hexane was poured into a 1-L glass funnel with a fritted disk. The gel was washed with 2 L of hexane prior to being loaded with 100 g of tocotrienol rich fraction from palm oil (containing 21% α-tocopherol) in 200 mL of hexane. The α-tocopherol was removed selectively by washing with a mixture of 10% ether in hexane (2 L), followed by washing with 30% ether in hexane (2 L), and the eluted fraction was evaporated under vacuum at 50°C, which yielded 66 g of tocotrienol rich fraction (TRF) free from α-tocopherol. The composition of various tocotrienols in this TRF was determined by high pressure liquid chromatography (HPLC) as described [41]. TRF was found to contain α-tocotrienol (15%), γ-tocotrienol (60%), and δ-tocotrienol (25%). TRF was stored in polyethylene glycol at -20°C at a concentration of 250 μg/mL. Plasma (40 mL) was warmed to 50°C (20 min) prior to injection. The α-Tocopherol (250 μg/mL), α-tocotrienol (250 μg/mL), or TRF (α- + γ- + δ-tocotrienols; 250 μg/mL) was dissolved in polyethylene glycol (1 mL of each) solution and added to 40 mL of warmed plasma individually, vortexed vigorously for 2 min, and then administered over 30 seconds through a femoral arterial line as reported previously [40]. The TRF used throughout present study is a mixture of α-, γ-, and δ-tocotrienols, free of α-tocopherol.

### *Ex vivo *platelet aggregation

Canine platelet aggregation was performed using a modification of a previously described human platelet methodology [18,40]. Blood (30 mL) was drawn through a femoral arterial line into 3.8% sodium citrate (1 citrate to 9 parts blood). Platelet-rich plasma (PRP) was isolated by centrifugation at 250 × g for 15 min. Platelet-rich plasma was separated and platelet counting was performed. The remaining blood was centrifuged further at 2,000 × g for 20 minutes and platelet-free plasma (PFP) isolated. PRP platelet counts were adjusted with corresponding PFP to equalize counts between pre- and post-treatment specimens. PRP counts varied between 100,000 and 200,000 platelets/microliter. Platelet aggregation was performed using a Sienco DP 247-E Dual Sample Aggregometer (Morrison CO) and Omniscribe recorder (Houston Instruments Model A5211-1), adjusting PRP to 10% transmittance and PFP to 90% transmittance.

Collagen (12.5 μmol/mL) or ADP (20 μmol/L), in volumes of 32 μL were added to PRP (400 μL), after the PRP was incubated at 37°C for 2 min. The extent of collagen-induced platelet aggregation was quantitated by measuring the percent aggregation 6 min after the addition of collagen. ADP-induced platelet aggregation was quantitated by measuring percent aggregation at both 2.5 and 6.0 min after adding ADP.

### Isolation and analyses of tocopherols and tocotrienols in plasma and platelets by HPLC

Blood (30 mL) was drawn in EDTA (0.8 parts 77 μM disodium EDTA to 9.2 parts blood) and PRP was prepared from pre-dose and post-dose specimens as described above. Platelets were separated from PRP by centrifugation at 1950 × g for 20 min and the platelet pellet washed twice with phosphate-buffered saline (PBS). The final platelet pellet was suspended in 1 mL PBS and platelets were counted. All samples were stored at -20°C until assayed.

Estimations of tocols (mixture of α-, β-, γ-, δ-tocopherol + α-, β-, γ-, δ-tocotrienols) in plasma and platelet fractions were carried out by HPLC using a modification of a previously-described methodology [41]. Plasma (2 mL) or platelets (1 mL) were added to a 20 mL culture tube, hexane (6 mL) was added, and these samples were shaken in a horizontal shaker for 20 min, followed by centrifugation at 1000 × g for 10 min. The hexane layer (free tocols) was transferred to a 15-mL conical tube and the solvent was removed under vacuum at 50°C in a vortex evaporator. The remaining aqueous layer was dried at 120°C in a vacuum oven (2 psi) for 60 min. The residue was extracted with methanol (6 mL) using a horizontal shaker for 30 min and centrifuged at 1000 × g for 15 min. The methanol soluble fraction (bound tocols) was separated and concentrated under vacuum at 50°C in a vortex evaporator. Tocols extracted by each solvent were dissolved in hexane (0.5 mL), vortexed and transferred to injecting vials (250 μL) the vials were capped and centrifuged at 1000 × g for 5 min, followed by HPLC analyses [41]. The tocols were eluted under these conditions in sequence; α-tocopherol (5.2 min), α-tocotrienol (5.6 min), β-tocopherol (6.8 min), β-tocotrienol (7.2 min), γ-tocopherol (7.8 min) γ-tocotrienol (8.1 min), δ-tocopherol (16.8 min) and δ-tocotrienol (18.4 min), respectively. Values of the four separate isomers of α-, β-, γ- and δ-tocopherols and tocotrienols were estimated separately, but combined values for the four isomers of tocopherols or tocotrienols were reported.

### Statistical analyses

The data were analyzed by using the GLM procedure of SAS (Statistical Analysis System) for personal computers to test the Duncan's multiple-range test to determine whether the post-dose treated values differed from the pre-dose values for α-tocopherol, α-tocotrienol, or TRF treated groups. A two-way ANOVA was used to test whether changes in collagen or ADP treated platelet aggregation occurs during the treatments and whether there were intra or inter- group differences between tocol levels. The differences between means were also evaluated using the paired, two-tailed student *t*-test (StarView, Abacus Concept) and data were reported as mean ± in the standard deviation. The level of statistical significance was reported at 5% (***P ***< 0.05).

## Results

### Effects of tocols on the levels of hematocrit and number of platelets (counts) in whole blood and platelet rich plasma (PRP)

In order to obtain an initial assessment of the effects of treatment with various tocols on factors potentially effecting thrombosis in dogs, we first measured the effects of these treatments on hematocrit and platelet counts. There were no significant differences between pre-dose and post-dose hematocrit levels after treatment with α-tocopherol (47.97 ± 6.36 vs 48.33 ± 0.64), α-tocotrienol (49.47 ± 1.87 vs 46.40 ± 2.99), or TRF (46.45 ± 5.84 vs 41.45 ± 7.01) as shown in Table 1. The cumulative average pre-dose and post-dose platelet counts in whole blood also did not change significantly after treatment with α-tocopherol (234.83 ± 42.09 vs 188.33 ± 54.47), α-tocotrienol (262.67 ± 49.61 vs 188.00 ± 36.23), or TRF (28.38 ± 63.25 vs 207.25 ± 43.25). In PRP, pre- and post-treatment platelet counts also did not change significantly with α-tocopherol (196.00 ± 6.93 vs 194.00 ± 6.00), α-tocotrienol (187.67 ± 10.79 vs 175.33 ± 10.79), and TRF (170.00 ± 24.49 vs 164.75 ± 17.42) thousand/μL (Table 1).

**Table 1:** Effects of tocols on hematocrit levels, and the number of platelets in whole blood and in platelet rich plasma in stenosed canine arteries

	**Hematocrit**^**1**^	
**Dogs**	**Pre-dose**	**Post-dose**

		
		
α-Tocopherol	47.97% ± 6.36 (100)^4^	48.33% ± 0.64 (101)
		
		
		
α-Tocotrienol	49.47% ± 1.87 (100)	46.40% ± 2.99 (94)
		
		
		
TRF^3^	46.45% ± 5.84 (100)	41.45% ± 7.01 (89)
		
		

		

	**Platelet Counts in Whole Blood**^**2**^	

**Dogs**	**Pre-dose**	**Post-dose**

		
		
α-Tocopherol	234.83 ± 42.09 (100)^4^	188.33 ± 54.47 (80)
		
		
		
α-Tocotrienol	262.67 ± 49.61 (100)	188.00 ± 36.23 (72)
		
		
		
TRF^3^	280.38 ± 63.25 (100)	207.25 ± 43.25 (74)

		

	**Platelet Counts in Platelet Rich Plasma**^**1**^	

**Dogs**	**Pre-dose**	**Post-dose**

		
		
α-Tocopherol	196.00 ± 6.93 (100)^4^	194.22 ± 6.00 (99)
		
		
		
α-Tocotrienol	187.67 ± 10.79 (100)	175.33 ± 10.79 (93)
		
		
		
TRF^3^	170.00 ± 24.49 (100)	164.75 ± 17.42 (97)
		
		

^1^Average of 3 or 4 treatments: α-Tocopherol (1 + 2 + 3); α-Tocotrienol

(4 + 5 + 6); TRF (mixture of α-, γ-, δ-tocotrienols; 7 + 8 + 9 + 10). Reported as thousands/μl.

^2^Average of duplicate analyses of 3 or 4 treatments: α-Tocopherol (1 - 3);

α-Tocotrienol (4 - 6); TRF (7 - 10). Reported as thousands/μl.

^3^TRF = tocotrienol rich fraction (α-tocotrienol + γ-tocotrienol + δ-tocotrienol)

^4^Percentages of pre-dose or post-dose values are in parentheses.

### Effects of tocols *in vivo*

As mentioned above, none of these compounds, when injected intravenously, had any significant effect on hematocrit levels or platelet counts in whole blood or in PRP, though small, non-significant decreases were observed (Table 1), which is characteristic of this model. We utilized the Folts' arterial injury model of stenosis and thrombosis because the effects of various compounds on platelet aggregation and platelet-mediated thrombus formation can be determined by measuring the cyclic flow reductions (CRFs) in mechanically stenosed circumflex coronary arteries (MSCCA). The size and frequency of these CFRs is directly related to the level of *in vivo *platelet aggregation activity [42,43]. Specifically, platelet-mediated thrombi form periodically in the stenosed coronary artery. This is followed by distal embolization, which produces cyclic reductions in measured coronary blood flow, known as cyclic flow reductions (CFRs) [42,43]. It has also been demonstrated that CFRs in mechanically stenosed canine arteries can be eliminated by platelet inhibitors (e.g. aspirin, prostacyclin, and the nitric oxide donor sodium nitroprusside) [44,45].

In the present study, there were no significant changes in blood pressure or heart rate during, or following, the intravenous (IV) administration of α-tocopherol, α-tocotrienol, or TRF. Importantly, however, the IV injection of α-tocopherol, α-tocotrienol or TRF consistently abolished cyclic flow reductions (CFRs) in mechanically stenosed circumflex coronary arteries of dogs. The average mean time between the IV injection of TRF (10 mg/kg) and the abolition of cyclic flow reduction (CFRs) was 68.4 ± 12 min (range 22-130 min). Once abolished, CFRs did not return to pre-dose values, despite challenge with epinephrine (standard drug for restoring CFRs to normal or pre-dose level) or increases in percent laminal stenosis. Monitoring for the return of CFRs to pre-dose levels was continued for as long as 160 min (range 50-160 min) post abolition of CFRs. Similar results were also obtained with IV of α-tocopherol (20 mg/kg), and α-tocotrienol (10 mg/kg) for CFRs. The average mean time between α-tocopherol (20 mg/kg), α-tocotrienol (10 mg/kg) or TRF (10 mg/kg; α- + γ- + δ-tocotrienols) intravenous administration and blood sample collection for determining platelet aggregation and tocol levels was 88 ± 10 min (41-117 min). The results of these studies support the concept that α-tocopherol, α-tocotrienol, and TRF all have protective effects against intravenous platelet aggregation and thrombus formation.

### Effects of α-tocopherol, α-tocotrienol or TRF on *ex vivo *platelet aggregation

**Figure 2:**
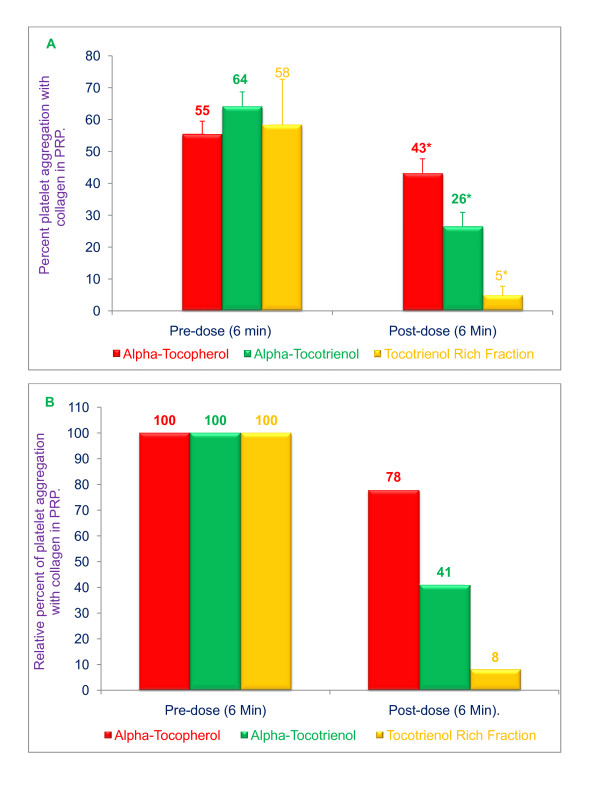
**Effects of α-tocopherol, α-tocotrienol, and tocotrienol rich fraction (TRF) on collagen-induced platelet aggregation**: α-Tocopherol (250 μg), α-tocotrienol (250 μg) or TRF (250 μg) were dissolved in polyethylene glycol (1 mL) solvent and stored at -20°C. Plasma (40 mL) was warmed to 50°C (20 min) prior to injection. α-Tocopherol, α-tocotrienol or TRF solutions in 1 mL of polyethylene glycol were added to warmed plasma, vortexed vigorously, and then administered over 30 seconds through a femoral arterial line to anesthetized dogs. Collagen (12.5 μmol/mL) in a volume of 32 μL was added to 400 μL of PRP that had been incubated at 37°C for 2 min. The extent of collagen-induced platelet aggregation was quantitated by measuring the percent maximal aggregation 6 min after adding collagen. Data are expressed as means ± SD, *n *= 3 (α-tocopherol), 3 (α-tocotrienol), and 4 (TRF) dogs respectively, per treatment. Figure 2A is based on raw values and 2B is based on percentages compared to their respective pre-dose and post-dose control values. An asterisk indicates significant differences at *P *< 0.001 for each treatment compared to respective controls.

In order to further evaluate the potential protective effects of tocols on platelet aggregation, we tested their effect on *ex vivo *collagen-induced platelet aggregation in PRP. Our results demonstrated that tocols had profound, and significant, effects on collagen-induced platelet aggregation. Platelet aggregation in PRP 6 minutes after treatment with α-tocopherol, α-tocotrienol, or TRF (mixture of pure tocotrienols only) was reduced by 22%, 59% and 92%, respectively (***P ***< 0.001; Figure 2). TRF was the most potent inhibitor amongst these compounds.

**Figure 3:**
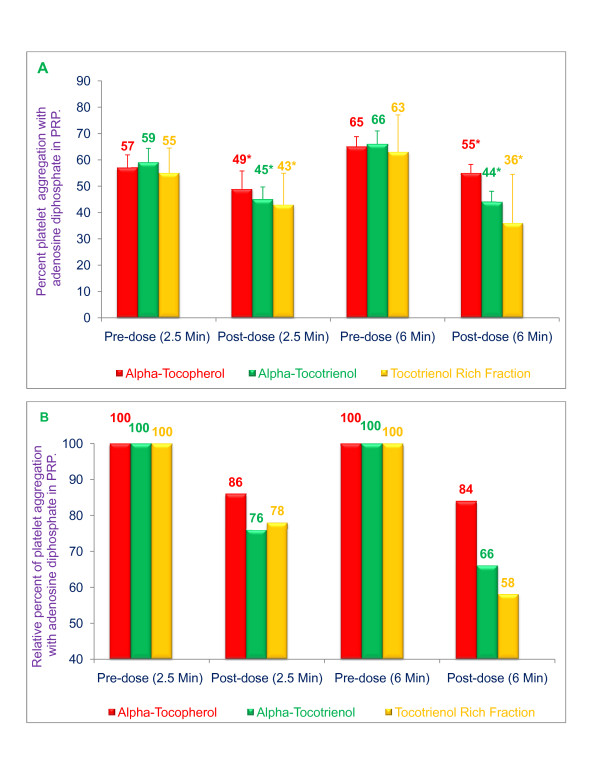
**Effects of α-tocopherol, α-tocotrienol, and tocotrienol rich fraction (TRF) on adenosine diphosphate-induced platelet aggregation**: α-Tocopherol (250 μg), α-tocotrienol (250 μg) or TRF (250 μg) were dissolved in polyethylene glycol (1 mL each) and stored at -20°C. Plasma (40 mL) was warmed to 50°C (20 min) prior to injection. The α-tocopherol, α-tocotrienol or TRF solution was added to the warmed plasma and vortexed vigorously, and then administered over 30 seconds through a femoral arterial line of the anesthetized dog. Adenosine diphosphate (ADP; 20 μmol/mL) in a volume of 32 μL was added to 400 μL of PRP that had been incubated at 37°C for 2 min. The extent of adenosine diphosphate -induced platelet aggregation was quantitated by measuring the percent maximal aggregation at 2.5 min and 6 min after adding ADP. Data are expressed as means ± SD, *n *= 3 (α-tocopherol), 3 (α-tocotrienol), and 4 (TRF) dogs respectively, per treatment. Figure 3A is based on raw values and 3B based on percentages compared to their respective pre-dose and post-dose control values. An asterisk indicates significant differences at *P *< 0.05 for each treatment compared to their respective control values.

Tocols (α-tocopherol, α-tocotrienol, or tocotrienols [α- + γ- + δ-tocotrienol]) also significantly inhibited adenosine diphosphate (ADP)-induced platelet aggregation in platelet rich plasma (PRP) as shown in Figure 3. Compared to pre-dose values, the extent to which platelet aggregation was inhibited at 2.5 and 6.0 min after the addition of ADP was 14% and 16% (***P ***< 0.05) for α-tocopherol, 24% and 34% (***P ***< 0.05) for α-tocotrienol, and 22% and 42% (***P ***< 0.025) for TRF (Figure 3). The maximal inhibition of platelet aggregation was, again, obtained with TRF (Figure 3).

### Effects of α-tocopherol, α-tocotrienol and TRF on the concentrations of tocols in plasma and platelets of dog

The concentrations of α-, β, γ, δ-tocopherols and α-, β-, γ-, δ-tocotrienols were estimated in plasma and platelets before injection and after CFRs were abolished. Following α-tocopherol treatment, tocopherol levels increased from 17.60 ± 0.32 to 56.91 ± 0.18 μg/mL (***P ***< 0.025) in pre-dose vs post-dose plasma specimens, respectively, and from 10.05 ± 0.27 to 42.22 ± 0.21 μg/mL in pre-dose vs post-dose platelets, respectively (***P ***< 0.025) as shown in Table 2. On the other hand, α-tocopherol treatment did not significantly change tocotrienol levels in plasma (2.89 to 1.49 μg/mL) or in platelets (2.84 to 2.33 μg/mL; Table 2). Following α-tocotrienol treatment, tocopherol levels increased from 10.10 ± 0.82 to 45.11 ± 2.19 μg/mL in pre-dose vs post-dose plasma specimens, respectively, and from 5.12 ± 1.24 to 28.16 ± 1.54 μg/mL in pre-dose vs post-dose platelets (***P ***< 0.025) as shown in Table 3. α-Tocotrienol treatment induced relatively modest increases in tocotrienol levels in plasma (0.90 ± 0.03 to 2.78 ± 0.13) and in platelets (1.02 ± 0.03 to 1.28 ± 0.04) as shown in Table 3. Pre-dose vs. post-dose levels of tocopherols and tocotrienols were also increased significantly with TRF treatment (tocopherols in plasma, 19.72 ± 1.29 to 67.38 ± 2.25 μg/mL; tocotrienols in plasma 1.65 ± 0.03 to 8.56 μg/mL ± 0.04; tocopherols in platelets, 7.85 ± 0.62 to 31.44 ± 1.84; tocotrienols in platelets, 3.85 ± 0.23 to 3.84 ± 0.12 μg/mL) as shown in Table 4.

**Table 2:** Effects of α-tocopherol on the concentration of tocols in canine plasma and platelets^1^

Treatments	**Total tocopherols (T)**^**2**^	**Total tocotrienols (T3)**^**3**^	Total T + T3
**α-Tocopherol (T)**		**Concentrations in μg/ml (ppm)**	

**Plasma (Pre-dose)**			
			
Total Tocols^4^	**17.60 ± 0.32**^**a **^**(86)**^**5**^	**2.89 ± 0.05**^**a **^**(14)**	**20.49 ± 0.26**^**a **^**(100)**

**Plasma (Post-dose)**			
			
Total Tocols	**56.91 ± 0.18**^**b **^**(97)**	**1.49 ± 0.03**^**b **^**(3)**	**58.40 ± 1.65**^**b **^**(100)**

**Platelets (Pre-dose)**			
			
Total Tocols	**10.05 ± 0.27**^**a **^**(80)**	**2.84 ± 0.02**^**a **^**(20)**	**12.89 ± 0.29**^**a **^**(100)**

**Platelets (Post-dose)**			
			
Total Tocols	**42.22 ± 0.21**^**b **^**(95)**	**2.33 ± 0.04**^**a **^**(5)**	**44.55 ± 0.22**^**b **^**(100)**

^1^Data expressed as means ± SD, *n *= 3, 3, and 4 respectively, per treatment.

^2^Total tocopherols = Mixture of α-tocopherol + β-tocopherol + γ-tocopherol + δ-tocopherol (T).

^3^Total tocotrienols = Mixture of α-tocotrienol + β-tocotrienol + γ-tocotrienol + δ-tocotrienol (T3).

^4^Tocols = Mixture of α-, β-, γ-, and δ-tocopherols (T) + α-, β-, γ-, and δ-tocotrienols (T3).

^5^Percentages of control values are in parentheses.

^a-b^Values in columns with a different superscript letter are significantly different at *P *< 0.05.

**Table 3:** Effects of α-tocotrienol on the concentration of tocols in canine plasma and platelets^1^

Treatments	**Total tocopherols (T)**^**2**^	**Total tocotrienols (T3)**^**3**^	Total T + T3
**α-Tocotrienol (T3)**		**Concentrations in μg/ml (ppm)**	

**Plasma (Pre-dose)**			
			
Total Tocols^4^	**10.10 ± 0.82**^**a **^**(92)**^**5**^	**0.90 ± 0.03**^**a **^**(8)**	**11.02 ± 0.81**^**a **^**(100)**
			

**Plasma (Post-dose)**			
			
Total Tocols	**45.11 ± 2.19**^**b **^**(94)**	**2.78 ± 0.13**^**b **^**(6)**	**47.89 ± 2.17**^**b **^**(100)**
			

**Platelets (Pre-dose)**			
			
Total Tocols	**5.12 ± 1.24**^**a **^**(83)**	**1.02 ± 0.03**^**a **^**(7)**	**6.14 ± 1.22**^**a **^**(100)**
			

**Platelets (Post-dose)**			
			
Total Tocols	**28.16 ± 1.54**^**b **^**(96)**	**1.28 ± 0.04**^**a **^**(4)**	**29.44 ± 1.52**^**b **^**(100)**


^1^Data expressed as means ± SD, *n *= 3, 3, and 4 respectively, per treatment.

^2^Total tocopherols = Mixture of α-tocopherol + β-tocopherol + γ-tocopherol + δ-tocopherol (T).

^3^Total tocotrienols = Mixture of α-tocotrienol + β-tocotrienol + γ-tocotrienol + δ-tocotrienol (T3).

^4^Tocols = Mixture of α-, β-, γ-, and δ-tocopherols (T) + α-, β-, γ-, and δ-tocotrienols (T3).

^5^Percentages of control values are in parentheses.

^a-b^Values in columns with a different superscript letter are significantly different at *P *< 0.05.

**Table 4:** Effects of TRF on the concentrations of tocols in canine plasma and platelets^1^

Treatments	**Total tocopherols (T)**^**2**^	**Total tocotrienols (T3)**^**3**^	Total T + T3
**TRF**^**4**^	**Concentrations in μg/mL (ppm)**		

**Plasma (Pre-dose)**			
			
Total Tocols^5^	**19.72 ± 1.29**^**a **^**(92)**^**6**^	**1.65 ± 0.03**^**a **^**(8)**	**21.37 ± 1.13**^**a **^**(100)**^**6**^
			

**Plasma (Post-dose)**			
			
Total Tocols	**67.38 ± 2.25**^**b **^**(89)**	**8.56 ± 0.04**^**b **^**(11)**	**75.94 ± 2.21**^**b **^**(100)**
			

**Platelets (Pre-dose)**			
			
Total Tocols	**7.85 ± 0.62**^**a **^**(67)**	**3.85 ± 0.23**^**a **^**(33)**	**11.70 ± 0.48**^**a **^**(100)**
			

**Platelets (Post-dose)**			
			
Total Tocols	**31.44 ± 1.84**^**b **^**(89)**	**3.84 ± 0.12**^**a **^**(11)**	**35.28 ± 1.70**^**b **^**(100)**

^1^Data expressed as means ± SD, n = 3, 3, and 4 respectively, per treatment.

^2^Total tocopherols = Mixture of α-tocopherol + β-tocopherol + γ-tocopherol + δ-tocopherol.

^3^Total tocotrienols = Mixture of α-tocotrienol + β-tocotrienol + γ-tocotrienol + δ-tocotrienol

^4^TRF = Tocotrienol Rich Fraction (Mixture of 15% α-tocotrienol + 60% γ-tocotrienol + 25% δ-tocotrienol)

from palm oil (Purified at AMR, Madison, WI).

^5^Tocols = Mixture of α-, β-, γ-, and δ-tocopherols (T) + α-, β-, γ-, and δ-tocotrienols (T3).

^6^Percentages of control values are in parentheses.

^a-b^Values in columns with a different superscript letter are significantly different at *P *< 0.05.

Although treatment with the various tocols had relatively modest effects on pre-dose vs. post-dose treatment levels of tocotrienols, these same treatments had much more profound effects on tocopherol levels (Tables 3, 4). After α-tocopherol treatment, total tocopherol concentrations increased approximately 3-fold in plasma and 4-fold in platelets (Table 2). Similarly, after treatment with α-tocotrienol and TRF, tocopherol levels increased more than a 4-fold and 5-fold, in plasma and platelets, respectively (Tables 3, 4). It has been demonstrated that administering γ-tocotrienol or TRF_25 _capsules to hypercholesterolemic humans for 4-weeks significantly increased serum α-tocopherol concentrations compared to baseline values [33,41]. This bioconversion was further confirmed by demonstrating the conversion of γ-(4-^3^H) tocotrienol to α-tocopherol *in vivo *using chickens [35,37,41].

## Discussion

Results of the present study clearly demonstrate the inhibitory effect of tocotrienols on *in vivo *platelet thrombus formation and *ex vivo *platelet aggregation using the Folt's stenosed canine coronary artery model. Significant and marked inhibition of collagen- or ADP-induced platelet aggregation was observed with α-tocotrienol and TRF (α-, + γ-, + δ-tocotrienols) treatments. This inhibition exceeded that achieved with α-tocopherol under similar conditions (Figs. 2, 3). This is the first report in which the effects of tocotrienols on platelet aggregation *in vivo *and *ex vivo *have been evaluated using the Folts' Model [40]. Although α-tocopherol, α-tocotrienol and TRF treatments were all effective in inhibiting collagen- and ADP-induced platelet aggregation in platelet rich plasma, the inhibition achieved with TRF was significantly greater than that observed with either α-tocopherol or α-tocotrienol. As mentioned earlier, the TRF used in this study contained α-, γ- and δ-tocotrienols (15%, 25%, and 60%, respectively), and was free of α-tocopherol.

Treatment with all of the tocols produced significant and pronounced increases in tocopherol concentrations in plasma and platelets. The effect of tocol treatments on tocotrienol levels was much more modest. It is important to recognize that α-tocopherol is the major form of vitamin E in plasma and platelets, even after treatment with pure α-tocotrienol or TRF_25_, due to bioconversion of tocotrienols to α-tocopherol [33-39,41]. The conversion of tocotrienols to α-tocopherol in plasma or platelets following IV injection in the current study suggests that uptake of tocotrienols by plasma or platelets is relatively rapid in the Folts model. To our knowledge, the time dependency of tocotrienol uptake in plasma or platelets has not been reported, and the results reported herein suggest that the majority of tocotrienols are ultimately converted to tocopherols as reported earlier [35,37,41], although the site of this conversion cannot be determined from the present data. Moreover, it is also unclear if the biological effects described above (i.e. decreased platelet aggregation, abrogation of CRF's) are secondary to tocotrienols, tocopherols, or a combination of both. The magnitude of the increase in plasma or platelet tocopherol concentrations in the present study is greater than that achieved in most dietary supplementation studies using α-tocopherol or tocotrienols, and it could be speculated that tocotrienols are utilized more efficiently than tocopherols by plasma or platelets. The increase in plasma tocopherols was also greater than that seen in most dietary supplementation studies using various formulations of α-tocopherol in TRF_25 _or Palmvitee capsules [35,37,41]. Whether the greater plasma and platelets levels of tocopherols in the present study are the result of a unique effect of tocotrienols, the intravenous route of administration, the formulation used (TRF_25_, Palmvitee), or an effect of improved recovery during assay of these vitamin E compounds, cannot be determined from these data.

Although, the present study does not delineate the mechanism of action of tocopherols or tocotrienols on platelet function, previous reports have suggested that α-tocopherol inhibits platelet thromboxane A_2 _production, increases vascular PGI_2 _production, inhibits the platelet release reaction, inhibits platelet calcium mobilization, alters platelet membrane fluidity, and inhibits platelet phospholipase A_2 _[46,47]_. _It has been also demonstrated that tocotrienols are highly effective at reducing expression of adhesion molecules on endothelial cells and inhibiting monocyte adhesion to endothelial cells [48].

Recently, it was reported that α-tocopherol inhibits platelet-mononuclear cell interaction, platelet aggregation and platelet protein kinase activity induced with either phorbol 12-myristate 13-actate or thrombin in humans [49]. Dietary supplementation of α-Tocopherol significantly inhibited the superoxide production, lipid oxidation, IL-1β secretion monocyte-endothelial cell due to inhibition of protein kinase activity [50]. In humans, α-Tocopherol also partially inhibits platelet protein kinase C (PKC), and this action of α-tocopherol on platelet function provides new insights into the anti-thrombotic and atherogenic properties of α-tocopherol [51].

Moreover, the mixture of α-, γ-, δ-tocopherols were more effective in preventing platelet aggregation as compared to α-tocopherol alone observed in humans [52]. This inhibition of platelet aggregation was associated with increased release of nitric oxide due to activation of endothelial constitutive nitric-oxide synthase and protein kinase C [52]. Similarly, the effects of α-tocopherol and γ-tocopherol differ with respect to low-density-lipoprotein oxidation, superoxide activity, platelet aggregation and arterial thrombogenesis in human studies [53]. γ-Tocopherol is more potent than α-tocopherol in these effects also [53]. However, most studies only show an effect in cultured cells or under *ex vivo *conditions. Importantly, cell culture studies are often conducted under conditions of vitamin E deficiency. This might partially explain the inconsistency observed between cell culture studies and studies performed in animals or humans [53].

Combined treatment of diabetic rats with α-tocopherol and acetylsalicyclic acid (aspirin) had a greater inhibitory effect on platelet aggregation, and reduced nitric oxide production, than either treatment alone [47,54]. Combination therapy also improved balance of thromboxane and prostacyclin compared to untreated diabetic rats [47,54]. Consequently, this combination therapy appears to induce beneficial physiologic changes that may protect tissues from detrimental thrombotic and ischemic phenomena [54]. It has previously been demonstrated that tocopherol levels in platelets are depressed in diabetic subjects and that these low levels may contribute to the increased incidence of atherosclerosis and thrombotic events in diabetic patients [55-57]. Supporting this concept is the demonstration that dietary α-tocopherol has been demonstrated to reverse abnormalities of platelet function in diabetic rats and patients [49-52]. The above mentioned properties and other positive biological effects of tocopherols have been reviewed comprehensive by Reiter et al. [58].

Recently, tocotrienols were found to be potent neuroprotective agents against stroke [31,59]. Specifically, incorporation of tocotrienols into the diet of hypertensive rats protected them against stroke-induced injury [31,59]. This protective property of tocotrienols was due its inhibition of pp66 (c-Src gene) kinase activation and 12-Lipoxygenase, which protect against glutamase- and stroke-induced neurodegeneration [60]. This protective effect of tocotrienols (in nanomolar concentrations) is independent of their antioxidant activity because tocopherols were effective only at higher (micromolar) concentrations [32]. Recently, it was reported that γ-tocotrienol was the most cardioprotective of all the isomers, followed by α- and δ-tocotrienols [61]. It was also suggested that, although these isomers possess comparable antioxidant properties, their abilities to potentiate signal transduction could be different [61]. On the other hand, our previous and recent findings showed that tocotrienols exhibit varying degrees of biological activity with δ-tocotrienol showing the most potency, followed by γ-tocotrienol, and then by α-tocotrienol [23,29]. The present results show that a mixture of tocotrienols containing mainly γ-, and δ-tocotrienol, is more potent than α-tocotrienol with respect to inhibition of collagen- or ADP-induced platelet aggregation. Further studies are required to clarify the potency of γ-tocotrienol vs δ-tocotrienol.

## Conclusions

Administration of intravenous tocotrienols markedly inhibits collagen and ADP-induced platelet aggregation, inhibits *in vivo *platelet thrombosis, and induces a 2-fold and 4-fold increase in plasma and platelet levels of α-tocopherol, respectively. These treatments induced less pronounced increases in plasma or platelet tocotrienols levels, suggesting that intravenous administered tocotrienols were ultimately converted to tocopherols in this model, comparable to results obtained in humans, hamsters, guinea pigs and swine [33-39,62-64]. The above results also suggest that tocotrienols and tocopherols may be important physiologically and therapeutically in the prevention of thrombotic events and atherosclerosis, and that tocotrienols can be used to supplement currently used antiplatelet agents. Aspirin and clopidogrel resistance are well-described phenomena [65,66], and the search for more effective anti-platelet agents is ongoing. The enhanced cholesterol-lowering properties of tocotrienols may make these compounds even more attractive than α-tocopherol as potential therapeutic or prophylactic agents for platelet inhibition and protection against myocardial infarction and stroke.

## Abbreviations

APTF: Acute Platelet-mediated Thrombus Formation; CFRs: Cyclic Flow Reductions; HPLC: High Pressure Liquid Chromatography; IV: intravenous; MSCCA: Mechanically Stenosed Circumflex Coronary Arteries; PA: Platelet Aggregation; PPP: Platelet Poor Plasma; PRP: Platelet Rich Plasma; T: Tocopherol; T3: Tocotrienol; Tocols: Mixture of α-, β-, γ-, δ-tocopherols + α-, β-, γ-, δ-tocotrienols; TRF: Tocotrienol rich fraction from palm oil (Mixture of 15% α-tocotrienol + 60% γ-tocotrienol + 25% δ-tocotrienol); TRF_25_: Tocotrienol rich fraction of rice bran (α-tocopherol 8.7%, α-tocotrienol 15.5%, β-tocotrienol 1.6%, γ-totrienol 39.4%, δ-tocopherol 4.4%, δ-tocotrienol 5.2%, *d*-desmethyl tocotrienol 20.9%, unidentified tocotrienols 4.3%).

## Competing interests

The authors declare that they have no competing interests.

### Authors' contributions

All the authors were involved in the design of the study. CWK carried out studies *in vivo *using dogs, and also performed *ex vivo *platelets aggregation studies using dog's samples in the laboratory of JDF. JDF is the pioneer in developing cyclic flow reductions (CFRs) model. CJP edited the manuscript. All the authors have read and approved the final version.
